# Mutation Screening of *ARR3*, *CACNA1F*, *P4HA2*, *TRPM1*, *COL2A1*, *COL11A1* and *PAX6* in a Chinese Cohort of 37 Patients with Early-Onset High Myopia

**DOI:** 10.3390/genes17040391

**Published:** 2026-03-29

**Authors:** Xue Liu, Huihui Chu, Yaru Sun, Haixia Zhao, Jifeng Yu

**Affiliations:** 1 Department of Ophthalmology, Beijing Children’s Hospital, Capital Medical University, National Center for Children’s Health, Key Laboratory of Major Diseases in Children, Ministry of Education, Beijing 100045, China; 2 Optometry and Refractive Center, The Affiliated Hospital of Inner Mongolia Medical University, Tongdao North Street, Hohhot 010050, China

**Keywords:** early-onset high myopia, pathogenic variants, likely pathogenic variants, whole exome sequencing

## Abstract

**Background/Objectives**: Early-onset high myopia (eoHM), defined as high myopia manifesting before 10 years of age, is largely attributed to genetic defects. This study aimed to investigate the genetic underpinnings of eoHM in a cohort of Chinese patients. **Methods**: We recruited 64 Chinese patients with eoHM. Comprehensive clinical evaluations were performed, and whole exome sequencing (WES) was conducted to identify potential pathogenic variants. The genetic findings were analyzed and correlated with the clinical phenotypes. **Results**: A total of 64 unrelated Chinese patients with suspected early-onset high myopia were initially recruited. Following whole exome sequencing (WES) and variant annotation, final 37 patients with variants in known myopia-associated genes were included in the analytical cohort. The mean age of onset for the cohort was 5 years (IQR, 4–7), with a mean spherical equivalent refraction of −7 D (IQR, (−8)–(−6)). Genetic analysis revealed variants in 28 known myopia-associated genes. We identified pathogenic or likely pathogenic variants in 11 of the 37 patients (29.7%, 95%CI: 0.1737–0.4590), while the overall diagnostic yield was 17.2% (11/64, 95%CI: 0.0970–0.2839) in initial 64 recruited patients. These genes included seven well-established eoHM-related genes, such as ARR3, CACNA1F, P4HA2, TRPM1, COL11A1, COL2A1, and PAX6. Additionally, variants of uncertain significance (VUS) in seven other candidate genes were detected in patients with eoHM. **Conclusions**: Our findings expand the genetic spectrum of eoHM and reinforce the critical role of genetic testing in its etiological diagnosis and clinical management. Observed patterns of genotype–phenotype associations are descriptive and should be considered hypothesis-generating, requiring validation in larger cohorts. Additionally, we identify several candidate genes that may serve as prospective biomarkers, though these findings require validation in larger cohorts and functional studies.

## 1. Introduction

Myopia is a common refractive disorder characterized by the focal point of light falling in front of the retina, leading to impaired distance vision. Based on spherical refraction error, myopia is categorized into mild (−0.50 D to −3.00 D), moderate (−3.00 D to −5.00 D or −6.00 D), and high myopia (HM ≤−6.00 D or axial length > 26 mm) [1]. The global prevalence of myopia is rising, with projections estimating that it will affect 49.8% of the world’s population by 2050; the proportion with high myopia is expected to increase from 2.7% to 9.8% [2]. In China, the prevalence of myopia among children and adolescents has reached approximately 52.7%, with the rate of high myopia also showing a significant upward trend, which is estimated to reach 17.6% by 2050 [3]. Notably, the onset of myopia is occurring at increasingly younger ages, leading to a growing number of cases progressing to severe HM. As a serious clinical and public health challenge, HM is associated with sight-threatening complications, including cataracts, choroidal atrophy, macular degeneration, retinal detachment, and glaucoma [4]. A thorough understanding of its pathogenesis is therefore essential. The etiology of HM is complex, involving both genetic and environmental factors, and remains incompletely understood. Accumulating evidence has demonstrated significant familial aggregation of HM, underscoring the important role of genetic factors in its development [5,6,7].

Early-onset high myopia (eoHM), defined as high myopia manifesting before the age of 10 years, is predominantly influenced by genetic factors [8]. To date, numerous studies have reported genetic variants associated with HM in eoHM patients, including autosomal dominant (AD) variants (SLC39A5, BSG, CCDC1, SCO2, ZNF644, P4HA2, CPSF1, TNFRSF21, NDUFAF7, DZIP1, XYLT1), autosomal recessive (AR) variants (CTSH, GRM6, LEPREL1, LRPAP1, LOXL3), and X-linked recessive (XLR) variants (ARR3, OPN1LW) [9,10]. Yang et al. identified 11 HM-related genes in eoHM patients without accompanying systemic complications [10]. Zhou et al. reported that approximately one-quarter of eoHM patients carried genetic variants associated with inherited retinal diseases (IRDs) [11]. More recently, a study by Rui et al. identified 22 gene variants in 28 patients with eoHM, six of which were linked to retinal dysfunction or Stickler syndrome [12]. Collectively, these findings reflect the considerable genetic heterogeneity and uncertain genotype–phenotype correlations in eoHM.

In this study, we aimed to descriptively characterize the genetic landscape of eoHM in a Chinese cohort, with the primary objective of identifying pathogenic variants and expanding the known mutation spectrum. As a descriptive genetic case series, this study presents observed genotype-phenotype patterns as preliminary findings that warrant confirmation in larger, well-powered cohorts. Furthermore, this study underscores the importance of systemic evaluation and long-term follow-up for patients with high myopia harboring mutations in syndrome-associated genes.

## 2. Materials and Methods

### 2.1. Patients

**Figure 1:**
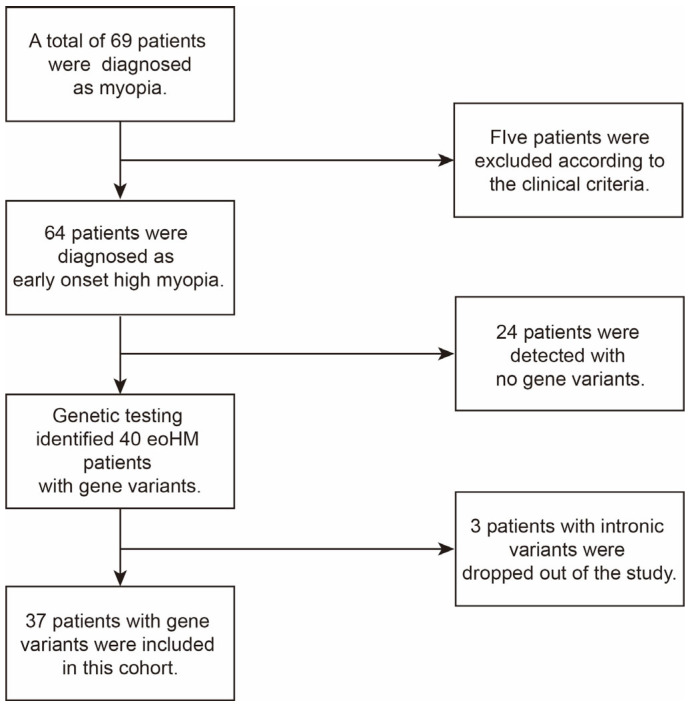
Flowchart of the study.

A total of 64 unrelated Chinese patients with suspected early-onset high myopia were initially recruited from the Ophthalmology Department of Beijing Children’s Hospital. The inclusion criteria were as follows: (1) spherical equivalent refraction (SER) ≤ −6.00 D or an axial length > 26 mm; (2) age of onset before 10 years; (3) absence of corneal diseases or other ophthalmic diseases that could cause high myopia; and (4) no congenital cataracts. Following WES and variant interpretation, we excluded 27 patients from the detailed genotype–phenotype correlation analysis for the following transparent reasons: 24 carried no variants in known myopia genes, and 3 harbored only deep intronic variants of uncertain significance. Clinical data were collected from medical records for all 37 participants. Due to the retrospective nature of this study, the primary available clinical parameter was SER. For patients with refractive data available for both eyes, the eye with the higher refractive error (i.e., more severe myopia) was selected for statistical analysis. The study flowchart is shown in Figure 1.

This study was conducted in accordance with the Helsinki Declaration and approved by the Ethics Committee of Beijing Children’s Hospital, Capital Medical University.

### 2.2. Whole Exome Sequencing (WES) and Variant Analysis

WES was performed for all patients to identify potential genetic causes of eoHM. Genomic DNA was extracted from peripheral blood samples using a Blood DNA extraction kit (Aowei Bio-Tech Co., Ltd., Foshan, China) according to the manufacturer’s instructions. Parental DNA was collected for segregation analysis when available (available for 3 of the 37 probands). After library construction, sequencing was carried out on the NovaSeq 6000 platform (Illumina, San Diego, CA, USA), achieving an average coverage of 100×, with over 98% of target bases covered at ≥20×. High-quality sequencing data were aligned to the GRCh38 reference genome (including the mitochondrial reference sequence NC_012920.1) using BWA software 2.2.1, and variant calling was performed by GATK 4.6.1.0. Subsequently, variants were annotated with ANNOVAR (https://annovar.openbioinformatics.org/en/latest/). Variants with a minor allele frequency ≤ 0.01 in population database were screened in databases, including gnomAD, ExAC, and 1000 Genomes project. Pathogenicity was predicted using SIFT (https://sift.bii.a-star.edu.sg/), REVEL (https://sites.google.com/site/revelgenomics/), the HGMD (http://www.hgmd.org/) and ClinVar (https://www.ncbi.nlm.nih.gov/clinvar/) databases (all accessed on 9 April 2025). The candidate variants that are associated with clinical phenotypes and aligned with the inherited patterns were classified as pathogenic (P), likely pathogenic (LP), or variant of uncertain significance (VUS) according to the American College of Medical Genetics and Genomics (ACMG) guidelines [13]. Subsequently, Sanger sequencing was performed to confirm these variants.

### 2.3. Statistical Analysis

Data were analyzed using SPSS 27.0 (IBM Corp., Armonk, NY, USA). The Shapiro–Wilk test was performed to assess the normality of data distribution. Continuous variables following a normal distribution are presented as means ± standard deviations (SD), whereas non-normally distributed variables are described using medians (interquartile ranges, IQRs).

## 3. Results

**Figure 2:**
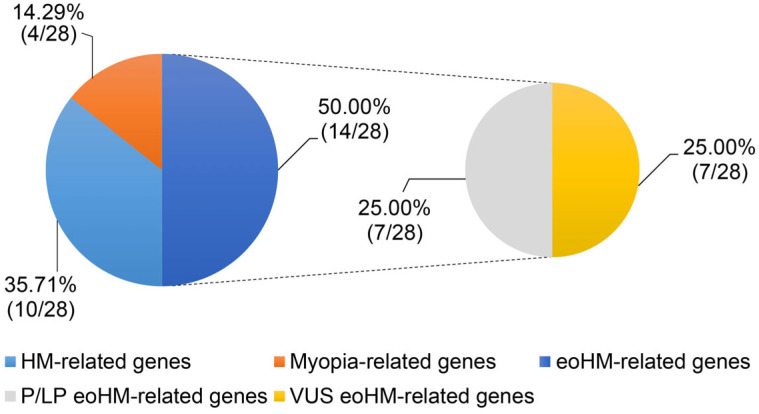
Percentage distribution of identified genes in the cohort.

A total of 64 unrelated Chinese patients with suspected early-onset high myopia were initially recruited, with the median onset age at 5 years (IQR, 3–7) and median spherical equivalent refraction −7 D (IQR, (−8)–(−6)). Of the 64 patients initially recruited, 37 patients carried at least one candidate variant in known myopia-associated genes and were included in the analytical cohort (24 females, 13 males). The median age of onset was 5 years (IQR, 4–7), and the median spherical equivalent refraction was −7 D (IQR, (−8)–(−6)). WES identified variants in 28 myopia-associated genes across all patients. Among these, 24 genes have previously been associated with high myopia. Pathogenic or likely pathogenic variants were confirmed in 11 of the 37 patients (29.7%, 95%CI: 0.1737–0.4590), involving seven eoHM-related genes: *ARR3*, *CACNA1F*, *P4HA2*, *TRPM1*, *COL11A1*, *COL2A1*, and *P*AX6. In the originally recruited cohort of 64 patients, the overall diagnostic yield was 17.2% (11/64, 95%CI: 0.0970–0.2839). Additional LP variants were identified in genes such as EYA1 and TGFBI; however, these genes were regarded as incidental findings as they did not align with the patients’ clinical phenotypes. The genotype and phenotype characteristics of all 37 patients are summarized in Appendix A Table A1. The distribution of identified genes is illustrated in Figure 2.

### 3.1. ARR3 Variants

We identified three patients with ARR3 variants, two of which were novel. ARR3, located at Xq13.1, comprises 17 exons and is specifically expressed in cone photoreceptors, playing a crucial role in color vision.

**Figure 3:**
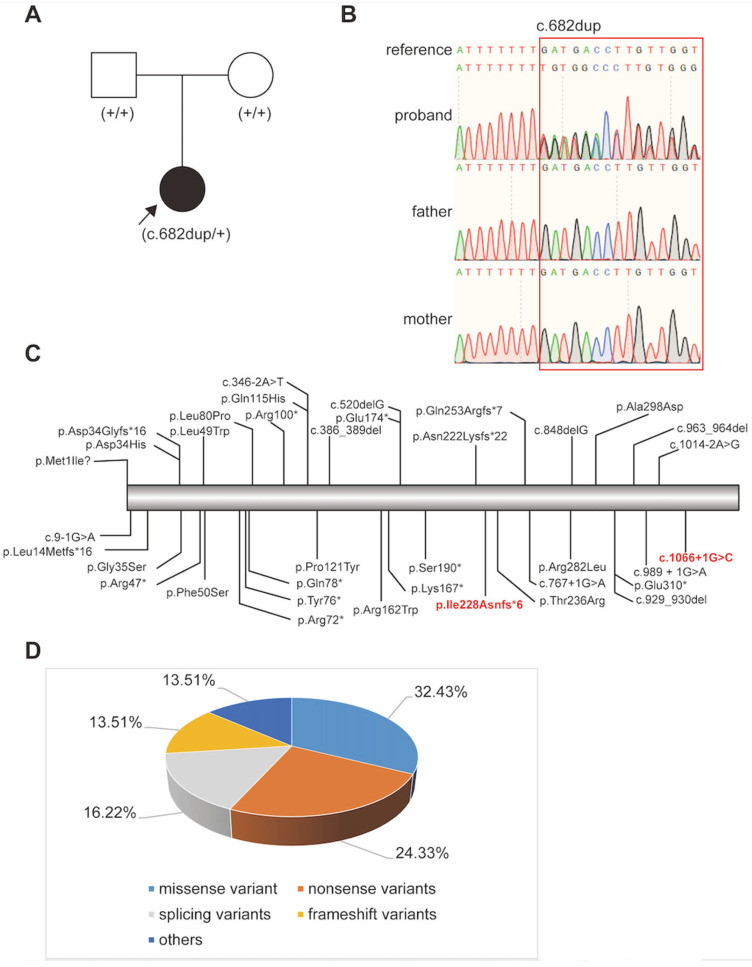
Genetic findings in Patient 2. (**A**) Family pedigree. The proband is indicated by an arrow. (**B**) Sanger sequencing confirmation of the de novo *ARR3* variant (c.682dup) in the patient and her parents. Green, red, black, and blue lines represent adenine (A), thymine (T), guanine (G), and Cytosine (C), respectively. (**C**) Schematic representation of all 37 reported ARR3 variants. The newly identified *ARR3* variants are highlighted in red font. The asterisk (*) indicates a premature stop codon, leading to a truncated protein. (**D**) Pie chart showing the proportion of different variant types in *ARR3*.

- Patient 1 carried a novel splice-site variant (NM_004312.3: c.1066+1G>C). This variant was absent from gnomAD and ExAC, and was not found in ClinVar. SpliceAI prediction indicated a high probability of splicing disruption (DS_DL = 0.91), suggesting impaired mRNA processing and potential loss of function. According to ACMG guidelines, the variant was classified as pathogenic (PVS1, PM2_Supporting, PP4).
- Patient 2 carried a de novo frameshift variant (NM_004312.3: c.682dup (p.Ile228Asnfs*6)), confirmed by Sanger sequencing (Figure 3A,B). This variant results in a substitution of isoleucine at position 228 to asparagine, followed by an extension of six aberrant amino acids before premature termination of translation. As the variant is not located within the last 50 bp of the terminal coding exon or the penultimate coding exon, it is predicted to trigger nonsense-mediated mRNA decay (NMD), potentially leading to loss of protein function. It was absent from gnomAD and was classified as pathogenic (PVS1, PM2_Supporting, PM6, PP4).
- Patient 3 carried a previously reported nonsense variant (c.298C>T (p.Arg100*)) associated with eoHM [14].

To date, 37 ARR3 variants have been reported in association with eoHM. As summarized in Figure 3C,D, missense variants are the most common (32.4%, 12/37), followed by nonsense (24.3%, 9/37), splicing (16.2%, 6/37), frameshift (13.5%, 5/37), and other types (13.5%, 5/37).

### 3.2. Pathogenic Variants in CACNA1F and P4HA2

Pathogenic variants were also identified in CACNA1F and P4HA2, both implicated in hereditary ocular disorders. Approximately 80% of patients with CACNA1F variants present with high myopia [15].

**Figure 4:**
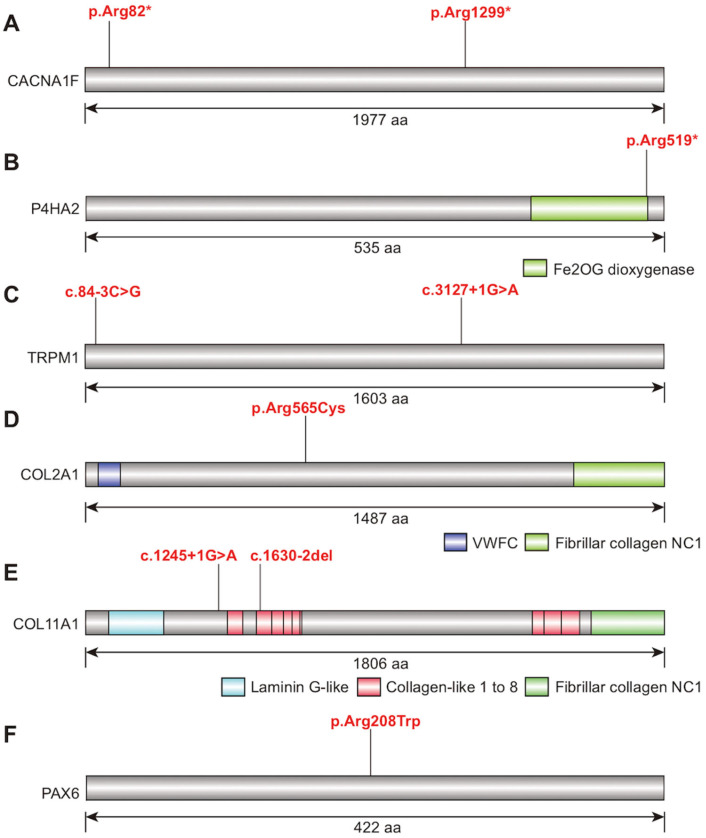
Schematic of protein structures showing the locations of identified pathogenic/likely pathogenic variants. (**A**) *CACNA1F*. (**B**) *P4HA2*. (**C**) *TRPM1*. (**D**) *COL2A1*. (**E**) *COL11A1*. (**F**) *PAX6*. The newly identified variants are highlighted in red. The asterisk (*) indicates a premature stop codon, leading to a truncated protein.

- Patient 4 carried a reported nonsense variant in CACNA1F (NM_005183.4: c.244C>T (p.Arg82*)) (Figure 4A), predicted deleterious by CADD (score = 35). It was classified as pathogenic (PVS1, PM2_Supporting, PS4_Moderate).
- Patient 5 carried a second nonsense variant (c.3895C>T (p.Arg1299*)) (Figure 4A), previously identified in individuals with myopia or ophthalmic lesions [16,17]. A high CADD score (45) supported its damaging effect, and it was rated as pathogenic.
- Patient 6 carried a P4HA2 nonsense variant (NM_001017974.2: c.1555C>T (p.Arg519*)) associated with autosomal dominant myopia (Figure 4B). This variant has been previously reported in eoHM [10], and its pathogenicity is supported by a high CADD score (41).

### 3.3. Potential Heterozygous Variants in TRPM1

We identified two TRPM1 splicing variants (NM_001252024.2: c.3127+1G>A and c.84-3C>G) in one patient (Figure 4C). TRPM1 is associated with autosomal recessive congenital stationary night blindness type 1C (CSNB1C). Although both variants affect splicing regions, their heterozygous status could not be confirmed due to lack of parental segregation data. The patient’s young age also precludes a conclusive assessment of whether these variants contribute to isolated high myopia or are part of a broader CSNB1C phenotype.

### 3.4. Syndrome-Associated Gene Variants

Pathogenic variants in COL2A1 and COL11A1 are typically linked to Stickler and Marshall syndromes, respectively.

- Patient 8 carried a COL2A1 missense variant (NM_001844.5: c.1693C>T (p.Arg565Cys)) (Figure 4D). This substitution occurs within the triple-helical domain and has been associated with ocular phenotypes. In silico predictions (REVEL, CADD) support a deleterious effect. However, it is important to note that a comprehensive systemic evaluation (including hearing, palatal, and musculoskeletal assessment) was not performed at baseline, as the initial referral was focused on the ophthalmic presentation.

Five patients carried COL11A1 variants, two of which were classified as pathogenic/likely pathogenic.

- Patient 12 carried a reported splice-site variant (c.1245+1G>A) (Figure 4E) [10], predicted by SpliceAI to strongly affect splicing (score = 1.0).
- Patient 13 carried a variant (c.1630-2del) that causes exon 15 skipping (Figure 4E) and has been detected in Stickler syndrome patients [11,18,19,20].

Although assessed as pathogenic/likely pathogenic per ACMG criteria, parental segregation analysis is required for definitive interpretation. Systemic evaluations for Marshall/Stickler syndrome features (e.g., hearing loss, facial dysmorphism, joint hypermobility) were not conducted at the time of initial diagnosis, representing a limitation of this retrospective study. These findings indicate that genetic screening for *COL11A1* in eoHM patients may aid in the early detection of syndromic disorders.

### 3.5. PAX6 Variant

We identified a previously reported *PAX6* variant (NM_000280.6: c.622C>T (p.Arg208Trp)) in Patient 15 (Figure 4F). This variant has been associated with myopia, isolated foveal hypoplasia, and other ocular abnormalities [21,22]. A high REVEL score (0.877) supports its deleterious effect, implicating *PAX6* as a potential genetic contributor to eoHM. While the patient presented only with ocular findings (myopia and suspected foveal hypoplasia), a full systemic evaluation for *PAX6*-related disorders (including neurological and endocrine assessments) was not performed.

## 4. Discussion

This study presents a comprehensive genetic analysis of 37 Chinese patients with early-onset high myopia (eoHM) through whole-exome sequencing. We identified pathogenic or likely pathogenic variants in seven eoHM-associated genes (*ARR3*, *CACNA1F*, *P4HA2*, *TRPM1*, *COL11A1*, *COL2A1*, and *PAX6*) in 11 patients (11/37, 29.7%, 95%CI: 0.1737–0.4590; 11/64, 17.2%, 95%CI: 0.0970–0.2839), thereby expanding the mutational spectrum of eoHM. Our findings, including two novel *ARR3* variants, contribute to the expanding genetic spectrum of ARR3 and highlight important diagnostic considerations. Furthermore, this study underscores the importance of systemic evaluation and long-term follow-up for patients with high myopia harboring mutations in syndrome-associated genes.

*ARR3*, specifically expressed in cone photoreceptors, is crucial for terminating phototransduction [23]. Prior studies have proposed several potential mechanisms by which ARR3 dysfunction could contribute to high myopia, including impaired cone function [24,25], a weakened retinal response to blue light [26], or dysfunction of the melanopsin system in ipRGCs [8]. *ARR3* variants typically exhibit X-linked female-limited inheritance [14,27]. Our identification of three *ARR3* variants, including a novel de novo frameshift variant, supports its inclusion in genetic screening panels, especially for female eoHM patients.

*CACNA1F*, another X-linked gene, is essential for retinal signal transmission. Variants disrupt calcium influx and glutamate release from photoreceptors and bipolar cells, impairing neural signaling [28,29]. Although classically associated with stationary night blindness, its involvement in isolated high myopia in our cohort illustrates significant phenotypic heterogeneity. These observations suggest that *CACNA1F* may warrant consideration even in the absence of classic night blindness, particularly in young patients, though the phenotypic spectrum of *CACNA1F*-related disorders in the context of isolated myopia requires further characterization.

*P4HA2* encodes a key enzyme in collagen hydroxylation [30]. As collagen is a major structural component of the sclera, defects in *P4HA2* are hypothesized to weaken scleral integrity, facilitating axial elongation [31,32]. The identification of a *P4HA2* nonsense variant in our cohort, consistent with reports across ethnic groups [10,31,33,34,35], strengthens its role in eoHM and suggests a common pathological mechanism involving collagen dysfunction. Importantly, emerging evidence indicates that EV-mediated intercellular communication plays a pivotal role in modulating extracellular matrix (ECM) composition and tissue remodeling [36]. Specifically, exosomes have been shown to transport miRNAs and proteins that regulate fibroblast activity, collagen synthesis, and ECM degradation, thereby influencing scleral biomechanics [37]. It is therefore plausible that P4HA2 deficiency in scleral fibroblasts may alter the cargo of secreted exosomes, which in turn could affect the behavior of adjacent fibroblasts or other cell types, contributing to aberrant scleral remodeling and progressive axial elongation.

The detection of *TRPM1* splicing variants in a young patient underscores a common diagnostic challenge in pediatric genetics. *TRPM1* is associated with CSNB1C [38], and variants have been reported in CSNB patients with high myopia [39,40]. However, the absence of nyctalopia in young patients does not rule out future onset [41]. Although compound heterozygosity was suspected but unconfirmed, this case emphasizes the importance of follow-up examinations when CSNB-related genes are identified in eoHM.

EoHM can be the earliest recognizable sign of potential Stickler syndrome (STL). We identified *COL11A1* or *COL2A1* variants in 7 patients (18.9%), suggesting these genes as potential contributors to STL-related high myopia. Notably, a significant proportion of eoHM patients with *COL11A1/COL2A1* variants do not meet the full clinical criteria for STL [42], and the rate of myopia progression in STL can be relatively low [43]. Our findings suggest the potential role of *COL2A1* and *COL11A1* as candidate molecular indicators for early identification, enabling timely multidisciplinary management.

Additionally, we identified VUS in several other genes previously implicated in eoHM (*ZNF644*, *SLC39A5*, *NYX*, *CPSF1*, *SCO2*, *GLRA2*, *COL9A3*). While classified as VUS, their recurrence in eoHM cohorts suggests potential modifier roles and warrants further investigation.

This study is a descriptive genetic case series that has several limitations. First, the small cohort size limited the statistical power for establishing definitive genotype-phenotype correlations. Second, the young age of many patients and the retrospective nature of the study limited the consistency of clinical phenotyping, with missing data for parameters such as axial length and systemic evaluations. Third, the absence of familial segregation data for several variants (e.g., *TRPM1*) and functional validation also constrains the interpretability of some findings. Fourth, we excluded patients with no mutations in known genes or those with only deep-intronic VUS. Although necessary for the current study, this may lead to an underestimation of the diagnostic yield in broader eoHM cohorts. Finally, as a single-center study, the findings may have limited generalizability. Consequently, our results should be viewed as a descriptive study. Future efforts should focus on expanding the sample size, incorporating longitudinal clinical assessments, and applying functional studies to validate the impact of novel variants.

## 5. Conclusions

In summary, our study highlights the genetic heterogeneity of eoHM and underscores the value of WES in its molecular diagnosis. We confirmed the role of seven known genes in eoHM, with *ARR3* and *CACNA1F* being the most frequently mutated in this cohort. The detection of novel variants broadens the mutational spectrum, and the identification of collagen gene mutations highlights a potential syndromic origin even in isolated ocular presentations. These findings support integrating genetic screening into the early diagnostic workflow for eoHM, enabling improved prognosis prediction, family counseling, and timely intervention. Furthermore, this study underscores the importance of systemic evaluation and long-term follow-up for patients with high myopia harboring mutations in syndrome-associated genes.

## Data Availability

The original contributions presented in this study are included in the article. Further inquiries can be directed to the corresponding authors.

## Abbreviations

The following abbreviations are used in this manuscript:

HM	high myopia
eoHM	early-onset high myopia
WES	whole exome sequencing

## Appendix A

**Table A1:** The genotype and phenotype characteristics of all 37 patients.

Patient ID	Gender	Onset Age	Spherical Refraction Error (Diopters (D))	Gene	Mutations	Protein Changes	Classification	ACMG Criteria Applied	gnomAD MAF
1	Female	5	−8	ARR3 *	c.1066+1G>C		P	PVS1+PM2_Supporting+PP4	\
2	Female	5	−7		c.682dup	(p.Ile228Asnfs*6)	P	PVS1+PM2_Supporting+PM6+PP4	\
3	Female	4	−7		c.298C>T	(p.Arg100*)	P	PVS1+PM2_Supporting+PP1_Strong+PP4	6.38 × 10^−6^
4	Male	7	−6	CACNA1F *	c.244C>T	(p.Arg82*)	P	PVS1+PM2_supporting+PS4_Moderate	5.46 × 10^−6^
5	Female	7	−8		c.3895C>T	(p.Arg1299*)	P	PVS1+PM2_supporting+PS4_Moderate	\
6	Male	4	−8	P4HA2 *	c.1555C>T	(p.Arg519*)	P	PVS1+PM2_Supporting+PP4	5.57 × 10^−5^
7	Female	8	−6	TRPM1 *	c.3127+1G>A		P	PVS1+PM2_supporting+PM3_supporting	5.60 × 10^−5^
					c.84-3C>G		VUS	PM2_supporting+PP3	1.21 × 10^−5^
8	Female	4	−8	COL2A1 *	c.1693C>T	(p.Arg565Cys)	P	PS2+PS4_Moderate+PM1+PM2_supporting+PP3	6.20 × 10^−7^
9	Female	7	−8		c.266G>A	(p.Cys89Tyr)	VUS	PM2_supporting+PP3	\
10	Female	3	−8	COL11A1 *	c.3545A>G	(p.Lys1182Arg)	VUS	PM2_Supporting+PP3	\
11	Female	4	−7		c.5274+4A>G		VUS	PM2_Supporting+PP3	3.99 × 10^−6^
12	Male	3	−6		c.1245+1G>A		LP	PVS1+PM2_supporting	1.62 × 10^−5^
13	Female	2	−8		c.1630-2del		P	PS3+PS4_Moderate+PM2_Supporting+PP1_Strong	6.84 × 10^−7^
14	Female	7	−8		c.2072G>A	(p.Gly691Asp)	VUS	PM2_Supporting+PP3	\
				COL5A1 *	c.3808G>A	(p.Gly1270Ser)	VUS	PM2_supporting+PP3	\
15	Female	4	−6	PAX6 *	c.622C>T	(p.Arg208Trp)	P	PS2+PM2_supporting+PP3+PS4	3.98 × 10^−6^
16	Male	4	−8		c.1124C>A	(p.Pro375Gln)	VUS	PM2_supporting+PP3	2.59 × 10^−4^
17	Female	2	−6	CRB1 $	c.2290C>T	(p.Arg764Cys)	P	PM2_supporting+PM1+PP3+PM3_very strong	7.57 × 10^−5^
18	Female	3	−7	FBN2 $	c.224G>A	(p.Arg75His)	VUS	PM2_supporting+PP3	6.21 × 10^−7^
19	Female	2	−6	NYX *	c.920A>G	(p.Asn307Ser)	VUS	PM2+PP3+PS4_supporting	8.28 × 10^−7^
20	Male	2	−10	ZNF644 *	c.3266A>G	(p.Tyr1089Cys)	VUS	PP3+PS4_Moderate	2.43 × 10^−4^
21	Female	6	−7		c.1648G>A	(p.Ala550Thr)	VUS	PM2_supporting+PP3	4.80 × 10^−5^
					c.829G>C	(p.Asp277His)	VUS	PM2_supporting+PP3	1.20 × 10^−5^
22	Male	5	−7	SLC39A5 *	c.648T>G	(p.Ser216Arg)	VUS	PM2_supporting	5.08 × 10^−6^
23	Male	8	−6		c.758C>A	(p.Ala253Glu)	VUS	PM2_Supporting+PP3+PS4_supporting	1.54 × 10^−5^
24	Male	5	−7	AGBL1 #	c.3271dupT	(p.Ala1093Cysfs*19)	VUS	PVS1_Moderate+PM2_Supporting	1.54 × 10^−4^
				SCO2 *	c.214G>C	(p.Gly72Arg)	VUS	PM2_supporting	6.22 × 10^−7^
25	Female	2	−6	TUBB3 #	c.845G>A	(p.Arg282Gln)	VUS	PM2_supporting+PP3	4.00 × 10^−6^
26	Female	5	−6	EYA1 #	c.1114dupA	(p.Thr372Asnfs*6)	LP	PVS1+PM2_Supporting	\
27	Female	5	−8	TGFBI $	c.1501C>A	(p.Pro501Thr)	LP	PS4+PP1+PP3	3.18 × 10^−4^
28	Male	5	−8	AIPL1 #	c.364G>A	(p.Gly122Arg)	VUS	PM2_supporting+PP3+PS3_supporting	\
29	Female	7	−6		c.256G>C	(p.Glu86Gln)	VUS	PM2_supporting+PP3	1.99 × 10^−5^
				CFI $	c.1006C>T	(p.Arg336*)	LP	PVS1+PM2_supporting	3.10 × 10^−6^
				HTRA1 $	c.1274C>T	(p.Ala425Val)	VUS	PM2_supporting	6.76 × 10^−5^
30	Female	8	−7	CPSF1 *	c.3980C>T	(p.Ser1327Leu)	VUS	PM2_supporting	4.51 × 10^−4^
31	Male	8	−7		c.2226C>G	(p.Asp742Glu)	VUS	PM2_supporting	5.14 × 10^−6^
32	Female	6	−7	PRIMPOL $	c.265T>G	(p.Tyr89Asp)	VUS	PS4_Moderate+PS3_Moderate	4.42 × 10^−4^
33	Male	7	−6	SAG $	c.68_75del	(p.Asp23Glyfs*27)	P	PVS1+PM2_supporting+PM3	8.03 × 10^−6^
34	Male	3	−6	EPHA2 $	c.1820A>C	(p.Glu607Ala)	VUS	PM2_supporting+PP3	2.78 × 10^−5^
35	Male	7	−8	GLRA2 *	c.754C>T	(p.Arg252Cys)	VUS	PM2_supporting+PP3	5.49 × 10^−6^
36	Female	3	−6	COL9A3 $	c.268C>T	(p.Arg90*)	LP	PVS1+PM2_Supporting	4.00 × 10^−6^
37	Female	3	−10	COL4A1 $	c.1238C>T	(p.Pro413Leu)	VUS	PM2_supporting+PP3	7.96 × 10^−6^

Note: *: genes associated with eoHM; #: genes associated with myopia; $: genes associated with HM.
